# Salivary Versus Circulating Insulin in Humans: Quantitative Evidence and Validation Requirements

**DOI:** 10.3390/biom16091348

**Published:** 2026-09-16

**Authors:** Lorenzo Albanese

**Affiliations:** Institute of BioEconomy, National Research Council of Italy, Via Madonna del Piano 10, 50019 Florence, Italy; lorenzo.albanese@cnr.it

**Keywords:** salivary insulin, circulating insulin, non-invasive biomarker, quantitative mapping, external validation

## Abstract

Salivary insulin offers a non-invasive approach to repeated metabolic assessment, but its quantitative relationship with circulating insulin remains incompletely defined. Paired human evidence was critically examined across the domains of cross-matrix association, temporal correspondence, scale, quantitative mapping, agreement, validation, and transferability. Salivary insulin tracked serum or plasma insulin under defined fasting and dynamic conditions, although association strength, temporal correspondence, and saliva-to-blood scale varied across populations, metabolic states, protocols, and salivary measurands. The available paired human evidence includes one explicit saliva-to-plasma mapping equation. Calibration, direct individual-level agreement, and independent external validation of a fixed mapping were not demonstrated, and individual prediction error was not characterized. Current evidence therefore supports salivary insulin as a context-dependent indicator of circulating insulin dynamics rather than a generally transferable quantitative substitute for serum or plasma insulin. Progression toward quantitative use requires explicit definition of the target measurand and temporal relationship, adequate analytical validation, calibration, characterization of individual prediction error, agreement assessment when quantitative substitution is intended, and independent external validation.

## 1. Introduction

Saliva can be collected non-invasively and repeatedly, supporting increasing interest in its use for endocrine and metabolic assessment [1,2]. These characteristics are particularly attractive when serial measurements are desirable, because sampling can be repeated with substantially less procedural burden than venous blood collection. Saliva has therefore been investigated for several decades as a diagnostic specimen for both local and systemic biomarkers [3,4]. Its practical accessibility, however, does not establish that the concentration of a circulating biomarker measured in saliva is quantitatively equivalent to that measured in blood.

The interpretation of a salivary measurement depends on the biological characteristics of the analyte and on the conditions under which the specimen is obtained and analyzed [5,6]. Routes of entry into saliva differ among molecular classes and can influence the meaning of the resulting measurement [7]. Collection, sample handling, and analytical procedures introduce additional sources of variability [8,9]. Relationships established for one salivary hormone therefore cannot be assumed to apply to another. For insulin, the saliva–blood relationship must be demonstrated directly rather than inferred from the general feasibility of salivary hormone measurement.

Interest in salivary insulin has developed within the broader search for non-invasive markers of metabolic status, obesity, and insulin resistance [10]. Paired human studies have also examined salivary insulin under fasting conditions and during glucose, meal, or insulin challenges [11,12]. These applications encompass different quantitative objectives. A salivary measurement may be useful for detecting variation or following the direction of an insulin response without necessarily reproducing the corresponding serum or plasma concentration. Estimating the circulating concentration or substituting a saliva-derived value for a blood measurement requires additional evidence.

This distinction is important because the published relationship between salivary and circulating insulin is not uniform. Strong cross-matrix correlations have been reported in some populations and experimental settings, whereas other studies have shown differences in association strength, timing, response magnitude, or individual correspondence [5,11,12]. Association, temporal correspondence, proportionality, quantitative mapping, calibration, prediction error, agreement, and transferability therefore represent different properties of the saliva-to-circulating-insulin relationship. Evidence supporting one property should not automatically be interpreted as evidence supporting another.

Recent reviews have considered salivary hormone diagnostics and endocrine or metabolic biomarkers from broader perspectives [1,2]. The narrower unresolved question is what can legitimately be inferred about circulating insulin from a salivary insulin measurement when the two matrices have been evaluated within physiologically linked human sampling. The objective was therefore to critically examine paired human findings according to the quantitative estimand addressed, clarify which levels of inference are currently supported, and define the validation required before quantitative estimation or substitution can be justified.

## 2. Scope and Evidence Framework

Quantitative human evidence was examined through a focused critical narrative synthesis of studies relating salivary insulin to serum or plasma insulin under physiologically linked paired sampling. Studies without a paired circulating-insulin comparator were used only to inform biological, sampling, or analytical context and not to support quantitative cross-matrix claims.

Relevant paired studies were located through targeted literature searches and reference chaining, with the paired-study literature considered through August 2026. The literature component was not designed as a systematic or scoping review; no review protocol was registered and no formal study-selection flow was constructed. Quantitative statements were limited to values explicitly reported in the original sources; missing values were not reconstructed or inferred.

Because the available studies addressed different quantitative questions, findings were interpreted according to the estimand evaluated, including cross-matrix association, temporal correspondence, proportionality or scale, quantitative mapping, agreement, and validation. Salivary insulin concentration and secretion output were treated as distinct measurands, and studies or publications derived from the same underlying cohort were not treated as independent replications. Because these quantities do not represent a single common saliva-to-circulating-insulin relationship, they were discussed separately rather than combined across studies.

## 3. Determinants of Quantitative Interpretation

### 3.1. Biological Origin and Salivary Measurand

The quantitative interpretation of salivary insulin first depends on what the measured signal represents biologically. Components detected in saliva can derive from circulating sources, local glandular processes, or combinations of both, depending on the analyte and physiological conditions [6,13]. The route by which a hormone enters saliva is therefore relevant to its interpretation [7]. Detection in saliva establishes the presence of an analyte-related signal, but does not by itself define its quantitative relationship with the corresponding circulating concentration.

Experimental insulin administration shows that salivary immunoreactivity can increase when circulating insulin is increased exogenously [14,15]. However, these studies did not establish assay-specific cross-reactivity with the administered insulin or molecularly identify the immunoreactivity detected in saliva. They therefore do not demonstrate that intact exogenous insulin reaches saliva or that saliva and blood assays recognize exogenous insulin equivalently.

Endogenous insulin reaches the systemic circulation after portal passage and partial hepatic extraction, whereas subcutaneously administered insulin does not undergo an initial hepatic first pass [16]. C-peptide, which is co-secreted with endogenous insulin but undergoes negligible hepatic extraction, provides a more direct marker of endogenous beta-cell secretion [17]. Salivary C-peptide has been measured in humans, but the cited study does not establish a quantitative saliva-to-circulating C-peptide relationship [18].

A salivary response does not imply proportional transfer. Entry into the oral fluid, salivary secretion, and the composition of the collected specimen can influence the magnitude of a salivary hormone signal [19]. A salivary response may therefore preserve information on the direction or temporal pattern of a circulating change without reproducing its absolute amplitude. Biological origin and quantitative correspondence should consequently be treated as related but distinct questions.

The definition of the salivary measurand introduces a second distinction. Salivary insulin concentration expresses the measured amount of insulin per unit volume of saliva, whereas secretion output additionally incorporates salivary flow. These quantities answer different questions. Concentration describes the composition of the collected fluid, while secretion output reflects the amount released over a defined period. A change in salivary flow can therefore alter the relation between concentration and output, even when both are derived from the same sampling interval.

This distinction is especially important when saliva is compared with serum or plasma insulin concentration. A circulating concentration-to-salivary concentration comparison and a circulating concentration-to-salivary output comparison do not estimate the same cross-matrix property. Results based on these different measurands should therefore remain separate rather than being interpreted as alternative estimates of a common saliva-to-blood ratio. Preserving the original measurand is necessary both for comparison among studies and for defining any future quantitative mapping.

### 3.2. Sampling and Temporal Construction

Saliva is not a single homogeneous specimen. Whole saliva contains contributions from the major and minor salivary glands together with components originating from the oral environment, whereas gland-specific collection isolates secretion from a more defined source [20,21]. The relative contribution of individual glands also varies with secretory conditions [21]. Whole saliva and gland-specific saliva should therefore be regarded as distinct sampling contexts when quantitative relationships with circulating insulin are compared.

Collection conditions further define the specimen being measured. Unstimulated and stimulated saliva differ in flow and glandular contribution, and several collection procedures are available, including passive drooling, expectoration, absorbent devices, and gland-specific methods [20]. Experimental evidence also shows that collection method can influence the measured level of some salivary biomarkers [22]. These effects are analyte-dependent and should not be used retrospectively to explain differences among insulin studies unless directly demonstrated. They nevertheless represent legitimate sources of methodological heterogeneity and should be reported when cross-study relationships are interpreted.

Hydration, recent food or drink, smoking, and acute stress can alter salivary flow or composition and therefore affect the collected specimen independently of circulating insulin [23,24,25,26]. These conditions should be standardized or recorded, including the interval since food or drink, when saliva–blood relationships are evaluated.

Salivary flow is particularly relevant because it influences the quantitative meaning of the measurement. Flow rate varies with stimulation and other physiological conditions [21]. Because concentration and secretion output incorporate flow differently, the two measurands should remain distinct throughout analysis. A relationship based on salivary output cannot be interpreted as a direct concentration-to-concentration comparison with serum or plasma insulin.

No validated salivary reference marker analogous to urinary creatinine is currently available for insulin normalization [27]. Salivary flow rate can be used to calculate analyte secretion output, but this changes the measurand from concentration to output rather than providing concentration normalization [23,28].

Food-related sensory stimulation can induce an early cephalic-phase insulin response, although its magnitude is variable [29]. The timing of food exposure should therefore be defined relative to saliva and blood sampling in basal and early postprandial protocols.

Temporal construction becomes equally important when insulin changes dynamically. After glucose ingestion, a meal, or exogenous insulin administration, circulating insulin may vary substantially over relatively short periods. A blood sample usually represents a discrete measurement, whereas a salivary sample may reflect secretion accumulated during a collection interval. Nominally matching sampling times therefore do not necessarily guarantee that saliva and blood represent identical physiological windows. Sampling interval and temporal alignment should consequently be considered part of the cross-matrix estimand rather than merely procedural details.

Dynamic responses have ranged from essentially simultaneous behavior to substantial temporal displacement under different experimental conditions [14,30]. In other protocols, the observed association changed when salivary measurements were shifted relative to circulating insulin [31,32]. Temporal alignment can therefore materially affect the apparent saliva–blood relationship.

A statistical time shift, however, identifies the alignment that best describes the observations in a particular dataset; it does not by itself establish a physiological transport delay. Observed peak displacement, differences in collection windows, response kinetics, and analytically optimized time shifting are distinct phenomena. For this reason, no fixed saliva-to-blood delay should be assumed in advance. Temporal correspondence should instead be established within the specific sampling design and population being studied.

### 3.3. Analytical and Data-Structure Determinants

A salivary insulin measurement can support cross-matrix interpretation only if analytical performance has been established in saliva. Sensitivity, precision, recovery, dilutional behavior, and matrix effects can affect absolute concentrations and apparent saliva–blood relationships [33,34]. Validation in serum or plasma does not establish equivalent analytical performance in saliva.

A commercially available insulin ELISA specifically designed and validated for saliva is available from Salimetrics [35].

The paired literature includes immunoreactive insulin as well as measurements distinguishing free and bound insulin [14,31]. These measurands are not necessarily interchangeable, and insulin immunoassays can differ in their cross-reactivity with recombinant human insulin and insulin analogues [36]. Assay specificity and the reported insulin measurand should therefore be preserved when interpreting studies involving exogenous insulin.

Study design introduces a separate statistical issue. Serial protocols commonly generate several saliva–blood pairs from the same participant, and these observations are correlated rather than independent. Treating each paired time point as though it came from a different participant can overstate the effective information contained in the dataset and produce misleading uncertainty estimates. Analyses of repeated cross-matrix observations should therefore preserve the participant-level clustering and distinguish within-participant covariation from between-participant evidence [37].

Repeated sampling can characterize a trajectory or temporal relationship, but it should not be confused with independent replication. A study with many paired observations from few participants may describe within-person dynamics in considerable detail while providing limited information on how the same relationship performs in new individuals. The number of observations, the number of participants, and the structure linking repeated measurements should therefore remain distinct when the strength and transferability of quantitative evidence are interpreted.

The principal biological, sampling, analytical, and data-structure determinants of cross-study comparability are summarized in Table 1.

Analytical comparability across paired studies is uneven. Saliva-specific assay performance and cross-reactivity with exogenous insulin were not consistently documented across the paired studies. Analytical heterogeneity should therefore be treated as a limitation of cross-study comparison rather than used to explain differences unless demonstrated within the relevant study.

## 4. Quantitative Human Evidence

The principal paired findings are summarized in Table 2 according to the quantitative estimand addressed. Because these estimands capture different properties of the cross-matrix relationship, their results should not be interpreted as interchangeable measures of a single quantitative relationship.

Taken together, the paired evidence supports context-dependent association and tracking, whereas support for transferable numerical conversion remains insufficient.

### 4.1. Cross-Matrix Association and Tracking

Cross-matrix association indicates that measurements in the two matrices tend to vary together within a defined population or experimental setting. Its interpretation depends on the study design: a fasting correlation describes between-participant covariation at a single physiological state, whereas association during a glucose or meal challenge may reflect repeated within-participant changes over time.

Fasting studies illustrate the range of relationships observed across populations. A strong correlation of r = 0.92 was reported in children, although the relationship weakened at higher circulating insulin concentrations [11]. Other populations showed more moderate, subgroup-dependent, or weak and non-significant associations [47,48]. A weak relationship was also observed in a clinical case–control population [49]. The collective evidence therefore supports fasting covariation under some conditions, rather than a uniformly strong saliva-to-blood association across populations.

Population-specific evidence remains limited and heterogeneous. Pediatric and obesity-related studies show variable association strength and temporal behavior [11,30,38]. Evidence in T1D and T2D is more limited and methodologically heterogeneous [15,30,31]. The available paired evidence does not support ranking population contexts by salivary-insulin performance or deriving a population-specific saliva-to-blood conversion.

Dynamic studies provide a different form of evidence. Salivary insulin has been shown to change in relation to circulating insulin during glucose, meal, and insulin challenges. In some protocols, strong associations were obtained after accounting for temporal displacement [31]. Other studies reported correspondence between integrated responses such as area under the curve [12,45]. These findings indicate that information can be retained across an evolving physiological response even when individual time points are not numerically equivalent.

The magnitude of a correlation should nevertheless be interpreted within the range and structure of the observations from which it was calculated. Correlation describes association rather than the differences between two measurements, and a high coefficient can coexist with systematic or individual discrepancies [50]. It therefore provides evidence that salivary and circulating insulin covary, but does not determine whether one measurement can reproduce, predict, or replace the other.

Strong association can support the interpretation that salivary insulin reflects variation in circulating insulin under the conditions studied. Quantitative estimation requires additional information on scale and prediction error, while substitution requires direct assessment of agreement. These questions are considered separately below rather than inferred from the strength of association alone.

### 4.2. Temporal Correspondence

Temporal correspondence addresses a different question from cross-matrix association: whether changes in salivary and circulating insulin are temporally aligned within a dynamic protocol. During dynamic challenges, a relationship may be present even if changes in the two matrices are not synchronized. The available paired evidence does not support a single fixed temporal relationship between salivary and circulating insulin.

Temporal displacement has been reported in several dynamic settings. In an early glucose-challenge study, the salivary response was displaced by approximately 45–90 min relative to circulating insulin [14]. An approximate displacement of 30–60 min was reported in children with obesity and insulin resistance during an OGTT [38]. In healthy adults exposed to glucose and meal challenges, the salivary response was displaced by approximately 30 min [41]. More recent repeated meal studies likewise reported temporal displacement of approximately 30–45 min [12]. These observations show that temporal displacement recurs across different experimental settings, but its magnitude is not constant.

Variation is evident even within a single experimental framework. During the same OGTT study, approximate peak displacement differed according to metabolic group: about 60 min in healthy participants, essentially no displacement in participants with type 2 diabetes, and about 30 min in obese non-diabetic participants [30]. This within-study contrast shows that temporal correspondence can vary even when the broad challenge protocol is shared. A single fixed temporal displacement therefore cannot be inferred from the available evidence.

Temporal adjustment can also affect the measured strength of association. In one healthy OGTT dataset, a strong cross-matrix correlation was obtained after a 30 min temporal adjustment [31]. In another meal-challenge study, the relationship became significant after a 60 min adjustment when circulating insulin was compared with salivary insulin output [32]. These results demonstrate that statistical alignment can reveal cross-matrix covariation that is not apparent from nominally simultaneous measurements.

Such adjustments should nevertheless remain distinct from directly observed response displacement. A time shift selected because it improves correlation identifies the temporal alignment that best fits a particular dataset; it does not establish the duration of insulin transfer from blood to saliva. Differences in response kinetics, sampling interval, collection duration, salivary measurand, and population can all contribute to the temporal pattern observed. The present evidence therefore supports context-dependent temporal correspondence, not a universal saliva-to-circulating-insulin lag.

### 4.3. Scale and Proportionality

Association describes whether salivary and circulating insulin vary together, whereas proportionality concerns whether their numerical relationship remains stable across the relevant measurement range. These properties are distinct. A strong association can occur even when the relative magnitude of the responses in saliva and blood differs across concentrations, physiological conditions, or populations.

Controlled hyperinsulinemia provides a within-study contrast. Circulating insulin increased from 176 ± 51 to 36,796 ± 2031 pmol/L, whereas salivary immunoreactive insulin increased from 253 ± 100 to 1919 ± 437 pmol/L [14]. Salivary insulin therefore responded clearly to the experimentally induced increase in circulating insulin, but the two matrices did not reproduce the same response amplitude.

Reported saliva-to-blood ratios provide a complementary description of scale. Their values are specific to the population, physiological condition, sampling design, concentration range, and analytical setting in which they were obtained [11,31,45]. A ratio derived from fasting measurements cannot therefore be assumed to apply to a dynamic postprandial response or to another population without separate evidence.

The salivary measurand further constrains interpretation. A relationship between circulating insulin concentration and salivary insulin secretion output does not estimate the same quantity as a concentration-to-concentration relationship. Concentration and secretion output should therefore remain separate when numerical scale is described, and relationships based on one measurand should not be treated as alternative estimates of the other.

The range over which a cross-matrix relationship has been characterized is also relevant. The weakening of fasting association at higher circulating insulin concentrations illustrates why a relationship observed over one range should not be extrapolated beyond that range without supporting evidence [11]. Demonstrating stable proportionality requires direct evaluation across the range and conditions relevant to the intended application rather than reliance on a single correlation coefficient or study-specific ratio.

Current paired evidence consequently supports study-specific descriptions of saliva-to-blood scale, but not a general proportional conversion between salivary and circulating insulin. Reported ratios remain descriptors of defined experimental conditions and should not be interpreted as transferable conversion factors.

### 4.4. Quantitative Mapping, Agreement, and Validation

A quantitative mapping goes beyond association by specifying an explicit numerical relationship between salivary and circulating insulin [44].

(1) $$\ln\;I_{P}=0.85\;\ln\;I_{S}+1.84$$

where $I_{P}$ and $I_{S}$ denote plasma and salivary insulin concentrations, respectively, expressed in pg/mL in the fitted relationship, and ln denotes the natural logarithm. The reported model fit for Equation (1) was R^2^ = 0.67.

Equation (1) shows that an explicit numerical mapping was fitted within the paired source dataset. It does not establish that the same relationship provides sufficiently accurate circulating-insulin estimates in new individuals. The reported R^2^ describes source-dataset model fit; it does not establish calibration or characterize individual prediction error [51].

Calibration and agreement address different quantitative properties. Calibration concerns the correspondence between predicted and observed values across the prediction range, whereas agreement concerns the magnitude and distribution of differences between paired values at the individual level [51,52,53]. A regression relationship can therefore show substantial association or model fit while individual discrepancies remain too large for numerical interchangeability.

Re-estimating a saliva-to-circulating-insulin relationship in another dataset may provide additional modeling evidence, but it does not constitute external validation of the original fixed mapping [54].

Current evidence therefore includes an explicit, dataset-specific saliva-to-plasma mapping, but not a validated general conversion between salivary and circulating insulin. Calibration, numerical agreement, internal validation, and independent external validation have not been demonstrated for a fixed saliva-to-circulating-insulin mapping, and individual prediction error has not been characterized. Quantitative mapping is therefore supported within a source dataset, whereas quantitative substitution and broader transferability remain unestablished.

## 5. Quantitative Evidence and Inferential Limits

Salivary insulin can covary with serum or plasma insulin under defined fasting and dynamic conditions, but the strength of this association varies across populations, metabolic states, protocols, and salivary measurands. Current evidence therefore supports cross-matrix tracking under specified conditions rather than a single quantitative relationship applicable across settings.

Temporal correspondence and numerical scale introduce additional context dependence. Dynamic studies show that salivary insulin can reflect changes in circulating insulin, but observed displacement varies across protocols and populations, including within a shared broad challenge framework. Statistical improvement after temporal shifting does not establish a physiological transport delay. Similarly, salivary insulin can respond clearly to changes in circulating insulin without reproducing the same response amplitude, and reported saliva-to-blood scale descriptors remain study-specific. Neither a universal temporal correction nor a general proportional conversion is therefore supported by the available evidence.

The reported saliva-to-plasma equation demonstrates that a numerical mapping can be fitted within a paired source dataset [44]. However, source-dataset model fit does not establish calibration, individual prediction accuracy, or numerical agreement [51,52].

Transferability consequently remains more restricted than cross-matrix association itself. A relationship demonstrated in one population, concentration range, sampling design, salivary measurand, or analytical setting cannot be assumed to retain the same timing, scale, or predictive performance in another. Association/tracking, temporal correspondence, scale/proportionality, quantitative mapping, calibration, agreement, validation, and transferability should therefore remain distinct quantitative domains, each supported only to the extent directly demonstrated by the available evidence. The current evidence status across these domains is summarized in Table 3.

**Figure 1:**
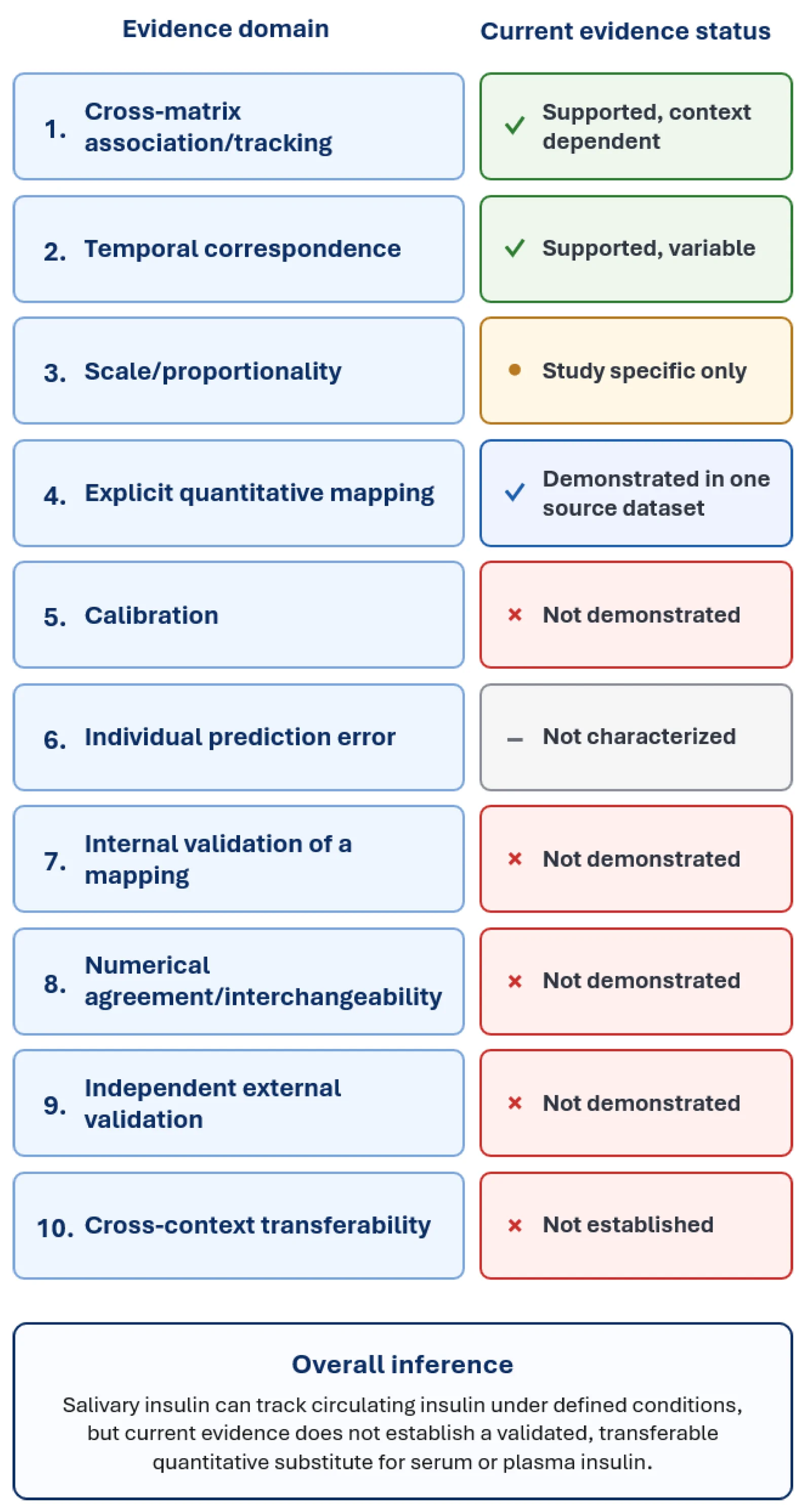
Current evidence status across quantitative domains of the salivary-to-circulating-insulin relationship. Symbols indicate evidence status: ✓, supported or demonstrated; •, study-specific evidence; ×, not demonstrated or not established; –, not characterized.

The domain-specific evidence status summarized in Table 3 is presented visually in Figure 1.

## 6. Validation Requirements for Quantitative Use

Progression from cross-matrix association to quantitative use requires validation matched to the specific inferential claim rather than a single generic demonstration of performance. This requires explicit definition of the target quantity and temporal relationship, analytical and statistical evaluation of the saliva-to-circulating-insulin relationship, and assessment of fixed-model performance in independent data.

### 6.1. Claim Definition and Temporal Design

Validation should begin with an explicit definition of the intended quantitative claim. Detecting whether salivary insulin changes in the same direction as circulating insulin, estimating a contemporaneous serum or plasma concentration, reconstructing a dynamic response, and substituting a saliva-derived value for a blood measurement represent different objectives and require different validation criteria [33]. Evidence sufficient for one objective should not be assumed to support another.

The circulating reference must therefore be specified as serum or plasma insulin, together with the analytical method, units, concentration range, and physiological condition in which the relationship is intended to operate. The salivary measurand must likewise remain explicit. Salivary insulin concentration and secretion output are not interchangeable quantities, and a model developed using one should not be applied to the other without separate validation.

Temporal definition is equally important for dynamic applications. The target may be a contemporaneous circulating value, a value corresponding to a predefined preceding time point, a peak response, or an integrated response over a specified interval. These targets represent different estimands. Sampling windows and any temporal displacement incorporated into a quantitative model should therefore be defined before performance is evaluated rather than selected retrospectively according to the shift that maximizes correlation.

Exploratory time-shifting can be useful during model development, but any resulting temporal rule should subsequently be fixed and evaluated in data not used to select it. Otherwise, apparent improvement in association may partly reflect optimization to the source dataset rather than a reproducible temporal relationship. Temporal design should consequently be treated as part of the quantitative model itself rather than as a secondary procedural detail.

Performance criteria should also be linked to intended use. A relationship intended only to follow the direction of an insulin response may tolerate greater individual discrepancy than one intended to estimate circulating insulin numerically. A substitution claim requires a predefined acceptance criterion appropriate to the intended use. Validation should therefore specify not only which statistical property is being evaluated, but also what level of performance would be considered acceptable.

### 6.2. Analytical and Statistical Validation

Quantitative cross-matrix validation requires adequate analytical performance within the salivary matrix before the saliva-to-blood relationship is assessed. Sensitivity, precision, recovery, dilutional behavior, matrix effects, and performance across the intended concentration range are relevant because analytical error in saliva contributes directly to uncertainty in any subsequent quantitative mapping [33,34]. Preanalytical procedures, including collection, processing, storage, and sample preparation, should likewise be sufficiently standardized to permit reproducible measurement [55].

Analytical validity, however, is only the first requirement. Cross-matrix association, calibration, individual prediction error, and numerical agreement answer different statistical questions and should be evaluated separately. Correlation can establish covariation but does not quantify the accuracy of predicted circulating values. Calibration evaluates whether predictions correspond appropriately to observed values across the prediction range, whereas prediction-error measures quantify the magnitude of individual estimation errors [51]. Agreement requires direct assessment of differences between paired values and should not be inferred from correlation or model fit [52].

When repeated saliva–blood measurements are obtained from the same participants, the dependence among observations must be preserved during both model development and performance assessment [37,53]. Treating repeated pairs as independent can exaggerate the effective sample size and can allow observations from the same participant to contribute to both model fitting and apparent validation. Participant-level separation is therefore required whenever observations are partitioned for performance evaluation.

A quantitative mapping should also distinguish model development from validation. Predictor definition, transformations, coefficients, temporal alignment, units, and other model components should be estimated within the development data and specified before validation. Apparent performance obtained from the same observations used to develop the relationship is expected to be optimistic to some degree and should not be interpreted as validated performance [54].

Internal validation can estimate this optimism when implemented appropriately: for example, through resampling or cross-validation procedures performed at the participant level and designed to preserve the dependence structure of repeated measurements. The complete model-development process relevant to the intended application should be represented within the validation procedure while preventing observations from the same participant from contributing simultaneously to model development and performance assessment. Calibration and individual prediction error should be reported alongside measures of association whenever numerical estimation is the intended claim.

If numerical substitution is proposed, agreement must be evaluated directly. Difference-based analyses should characterize systematic bias and the distribution of individual differences, using methods appropriate to repeated measurements when applicable [52,53]. A statistically strong regression relationship is insufficient if the resulting discrepancies remain too large for the intended use. Acceptable error should therefore be defined prospectively according to the intended application rather than inferred after the observed performance is known.

### 6.3. External Validation and Transferability

External validation requires evaluation of a previously developed relationship in participants who did not contribute to model development. The model should be applied in its fixed form, including the original coefficients, transformations, units, salivary measurand, and temporal definition [54]. Re-estimating coefficients in the new dataset produces a new model and therefore does not demonstrate the external performance of the original mapping.

A randomly separated subset of the same source cohort may provide useful internal performance information, but it does not provide the same evidence as evaluation in a genuinely independent paired cohort. Independent external validation should determine whether the fixed relationship retains adequate calibration and acceptable individual prediction error under the intended conditions of use [54]. Where numerical substitution is proposed, agreement with the directly measured circulating reference should also be assessed in the validation cohort.

External performance should be interpreted within the context in which it was obtained. Validation in one population or protocol establishes neither universal performance nor unrestricted portability. Differences in age, metabolic state, insulin range, fasting or postprandial condition, saliva collection, salivary flow, assay platform, specimen handling, and temporal design may alter the relationship between matrices. Transferability therefore requires evidence across the specific contexts included in the intended claim.

Successful validation of a fixed mapping in an independent cohort would initially support only the conditions represented by that cohort. Broader quantitative use would require replicated external validation across additional populations, analytical settings, and physiological conditions.

Compared with saliva, dried blood spots and volumetric absorptive microsampling retain blood as the sampled matrix and therefore avoid the need for saliva-to-blood inference, although matrix-specific analytical validation remains necessary [56,57]. Interstitial-fluid approaches permit serial or wearable sampling but also require insulin-specific validation because interstitial and circulating concentrations are not directly interchangeable [58,59].

The validation pathway should therefore remain claim-specific. Association supports covariation; temporal validation supports a defined timing relationship; calibration and prediction-error assessment support numerical estimation; agreement assessment can support an interchangeability claim only when evaluated against a predefined acceptance criterion; and external validation establishes the performance of a fixed relationship in new data. None of these properties should be inferred solely from another. For progression toward quantitative use, further demonstrations of cross-matrix association alone are insufficient; pre-specified, fixed saliva-to-circulating-insulin relationships require evaluation through calibration, characterization of individual prediction error, agreement assessment when quantitative substitution is intended, and independent external validation. The minimum requirements associated with each quantitative claim are summarized in Table 4.

## 7. Limitations

The available evidence is heterogeneous with respect to population, metabolic condition, saliva collection procedure, salivary measurand, temporal design, analytical method, concentration range, and statistical estimand. These differences limit direct comparison across studies and preclude interpreting the literature as estimating a single saliva-to-circulating-insulin relationship. Findings should therefore remain tied to the experimental and physiological conditions under which they were obtained. Because the literature synthesis was narrative rather than systematic, selection bias cannot be excluded, no formal risk-of-bias assessment was performed, and no pooled effect estimate was calculated.

Several dynamic investigations involved repeated saliva–blood measurements, whereas other studies were based on single paired assessments. Repeated observations can characterize within-participant trajectories but do not constitute independent replication across individuals. In addition, sample size, participant structure, and handling of repeated measurements were not always reported with sufficient detail to permit uniform statistical interpretation.

The available evidence includes assessments of detectability, cross-matrix association, and response tracking, but not evaluations of calibration, individual prediction error, numerical agreement, or external validation. The absence of these evaluations should therefore be interpreted as absence of evidence for the corresponding quantitative claim, not as evidence that satisfactory performance is impossible. Quantitative mapping evidence also remains limited to a source-dataset relationship without validation of fixed-model performance. Conclusions concerning numerical estimation, substitution, and broader transferability must consequently remain more restricted than those concerning association or dynamic tracking.

## 8. Conclusions

Salivary insulin can track variation in circulating insulin under defined human conditions. However, the relationship is context-dependent: association strength, temporal correspondence, cross-matrix scale, and quantitative interpretation vary with population, metabolic condition, sampling design, and salivary measurand. Current evidence therefore does not support a universal saliva-to-serum or saliva-to-plasma conversion.

An explicit saliva-to-plasma mapping has been reported, but its calibration has not been established, individual prediction error has not been characterized, and direct numerical agreement and independent external validation of the fixed relationship remain unestablished. Salivary insulin should therefore currently be regarded as a potentially informative non-invasive indicator of circulating insulin dynamics rather than a generally transferable quantitative substitute for serum or plasma insulin. Progression toward quantitative use requires explicit definition of the target measurand and temporal relationship, adequate analytical validation, calibration, characterization of individual prediction error, agreement assessment when quantitative substitution is intended, and independent external validation.

## Figures and Tables

**Table 1:** Study-level analytical and design determinants of cross-matrix comparability.

Population and Protocol	Paired Sample and Temporal Design	Circulating Reference and Assay	Salivary Measurand and Assay	Primary Comparability Feature
Healthy adults; OGTT and euglycemic IV insulin clamp [14]	OGTT *n* = 5; clamp *n* = 5; serial paired sampling	Serum/plasma insulin; RIA	Stimulated salivary IRI concentration; RIA with immunoreactive-insulin characterization	RIA was reported for both matrices; two experimental groups and distinct protocols were used, and the two matrices did not show the same relative response amplitude.
Healthy, T2D, and obese non-diabetic adults; OGTT [30]	*n* = 20; three metabolic subgroups; serial OGTT	Plasma insulin concentration; RIA	Whole-saliva insulin secretion output; RIA	RIA was reported for both matrices, but circulating concentration and salivary secretion output are non-commensurate; subgroup structure must be preserved.
Children/ adolescents with obesity and insulin resistance; OGTT [38]	*n* = 31; repeated paired OGTT sampling	Serum insulin; RIA	Salivary IRI; RIA; collection mode NR	RIA was reported for both matrices; the saliva-collection mode was not reported in the source.
Healthy adults; OGTT; T1D participants; daytime profiles [31]	Healthy *n* = 8; T1D *n* = 12; serial sampling in separate participant groups	Serum insulin; immunoassay; free/bound fractions characterized in T1D	Salivary insulin; immunoassay; free/bound fractions characterized in T1D	Participant groups, protocols, and insulin fractions are distinct. Cross-reactivity with exogenous insulin was not reported.
Non-diabetic participants; fasting [39]	*n* = 93; single fasting paired measurement	Plasma insulin; RIA	Salivary IRI output; RIA; salivary assay sensitivity, precision, and recovery characterized	RIA was reported for both matrices and analytical performance in saliva was characterized; the design was limited to a single fasting measurement.
Adults; OGTT [40]	*n* = 20; serial paired sampling	Serum/plasma insulin; assay NR	Salivary insulin; assay NR	Analytical and other methodological details were limited in the source.
Insulin-treated diabetes; fasting and post-injection experiment [15]	*n* = 45 fasting; nested *n* = 6 dynamic subset	Plasma free insulin; RIA after extraction	Salivary IRI concentration; direct RIA	Analytical handling differed between matrices. Cross-reactivity with exogenous insulin was not reported; *n* = 6 applies only to the nested dynamic experiment.
Healthy adults; oral-glucose and test-meal challenges [41]	*n* = 9; crossover cohort; repeated paired sampling	Plasma insulin concentration; immunoreactive- insulin assay	Salivary insulin secretion output; immunoreactive-insulin assay	Immunoreactive-insulin assays were reported for both matrices, but circulating concentration and salivary output are non-commensurate; repeated observations derive from the same participants.
Healthy adults; swallowed versus sham-fed meal crossover [42]	*n* = 12; serial within-participant sampling	Plasma insulin concentration; immunoreactive- insulin assay	Whole-saliva insulin concentration; immunoreactive-insulin assay	Immunoreactive-insulin assays were reported for both matrices; crossover conditions and repeated observations within participants are not independent.
Women with bulimia nervosa and matched controls; meal challenge [32]	*n* = 40; repeated postprandial sampling	Serum insulin; assay NR	Whole-saliva insulin concentration and secretion output; assay NR	Assay details were not reported in the source. Concentration and secretion output are distinct measurands; simultaneous and time-shifted comparisons are distinct estimands.
Separate adult and pediatric PCOS-related cohorts; OGTT [43]	Adults *n* = 30; pediatric cohort *n* = 32 at baseline and *n* = 30 at 2 h	Serum insulin; serum insulin assay	Salivary insulin; salivary insulin assay	Two independent cohorts differ in population and temporal resolution; analytical equivalence between the serum and salivary assays was not established.
Children aged 6–14 years; fasting [11]	*n* = 277; single fasting paired measurement	Serum insulin; automated chemiluminescence	Salivary insulin concentration; method based on the same analytical principle and specifically validated for saliva	The same analytical principle was used across matrices and the salivary method was specifically validated; the design was limited to a single fasting measurement.
Adolescents; paired assessment [44]	*n* = 53; single paired sample	Plasma insulin; multiplex assay	Salivary insulin concentration; multiplex assay	Multiplex assays were reported for both matrices; quantitative mapping was developed within the same paired source dataset.
Normal-weight and overweight/obese adults; low- versus high-carbohydrate meal crossover [45]	*n* = 16 completed; serial paired sampling	Plasma insulin; insulin immunoassay	Salivary insulin concentration; salivary insulin immunoassay	Both meal conditions derive from the same participants; analytical equivalence between the plasma and salivary immunoassays was not established.
Children with obesity ± hepatic steatosis and normal-weight controls [46]	*n* = 41; single paired clinical assessment	Serum insulin; clinical insulin assay	Salivary insulin concentration; salivary assay	Single-time-point pediatric clinical design; analytical equivalence between the serum and salivary assays was not established.
Healthy children aged 8–12 years [47]	*n* = 129; single paired assessment	Serum insulin; clinical assay	Salivary insulin; salivary assay	Cross-sectional pediatric design with subgroup-specific analyses; analytical equivalence between the serum and salivary assays was not established.
Adolescents; fasting [48]	Single fasting paired assessment; exact insulin-specific paired *n* not separately reported	Serum insulin; 25 µL multiplex magnetic-bead panel, Luminex 200	Unstimulated salivary insulin; 25 µL multiplex magnetic-bead panel, Luminex 200	The same analytical platform and panel were reported for both matrices; the insulin-specific pairwise denominator must not be inferred.
Acne vulgaris patients and controls; fasting [49]	*n* = 120; single fasting paired assessment	Serum insulin; ELISA/clinical assay	Salivary insulin; ELISA/clinical assay	ELISA/clinical assay was reported for both matrices; single-time-point case–control design.
Normal-weight and overweight/obese adults; two randomized meal crossover studies [12]	Two independent cohorts, *n* = 8 each; serial paired sampling over 9 h	Plasma insulin; plasma insulin assay	Salivary insulin concentration; salivary insulin assay	Cohorts are independent; repeated observations and crossover conditions within each cohort are not. Analytical equivalence between the plasma and salivary assays was not established.

Note: IRI, immunoreactive insulin; NR, not reported in the source; OGTT, oral glucose tolerance test; PCOS, polycystic ovary syndrome; RIA, radioimmunoassay; T1D, type 1 diabetes; T2D, type 2 diabetes. *n* denotes participants contributing to the stated protocol or cohort; repeated observations are not counted as independent participants. Where protocol-specific denominators differ, they are reported separately. Assay descriptions are retained only to the level documented in the source. Reporting the same nominal assay class or analytical platform across matrices does not by itself establish identical antibody specificity, calibration, analytical sensitivity, matrix performance, or cross-reactivity. Cross-reactivity with exogenous insulin was not inferred in the absence of explicit reporting.

**Table 2:** Quantitative paired-human evidence organized by estimand.

Quantitative Estimand/ Evidence Cluster	Verified Quantitative Evidence	Supported Inference	Inferential Boundary
Fasting cross-matrix association—stronger reported associations	Fasting salivary IRI output: men r = 0.75 and women r = 0.72 (both *p* < 0.0001) [39]. Fasting salivary insulin concentration in children: r = 0.92 (*p* < 0.001); at serum insulin >20 μIU/mL, r = 0.57 (*p* = 0.083) [11].	Strong fasting cross-matrix covariation can occur under defined conditions.	Association strength is context- and range-dependent; salivary output and concentration are distinct measurands, and correlation does not establish numerical agreement.
Fasting cross-matrix association— heterogeneous/weaker findings	Girls r = 0.442 (*p* < 0.05), with no significant association overall or in boys [47]; r = 0.40 (*p* < 0.0001) [48]; r = 0.2042 (*p* = 0.11) [49].	Fasting association varies across populations and subgroups.	No universal fasting association coefficient is supported. The exact insulin-specific pairwise denominator was not separately reported in the source [48].
Dynamic cross-matrix association	Healthy OGTT: serum insulin versus salivary insulin measured 30 min later, r = 0.810 (*p* < 0.01) [31]. Repeated glucose/meal challenges: plasma–salivary insulin association r = 0.74, with salivary insulin reported as output [41].	Dynamic cross-matrix association can occur under defined repeated-sampling protocols.	The 30 min result is temporally adjusted rather than contemporaneous; salivary output is not equivalent to concentration, and neither result establishes numerical agreement.
Integrated dynamic association	Adult 2 h insulin AUC r = 0.67 (*p* < 0.0001) [43]; 2 h AUC r = 0.821 (LC) and r = 0.882 (HC) (both *p* < 0.001) [45]; 9 h AUC r = 0.68 (HC, *p* = 0.007) and r = 0.84 (LC, *p* < 0.001) [12].	Integrated salivary and circulating insulin responses can show cross-matrix association under defined challenge conditions.	AUCs from different protocols and integration periods are not pooled; integrated association does not provide pointwise conversion or numerical agreement.
Measurand- and timing-dependent association	Simultaneous blood–saliva insulin concentrations were not significantly associated; blood insulin concentration versus salivary insulin output after a 60 min adjustment was significant (*p* = 0.018) [32].	The observed association can change when the comparison definition changes in salivary measurand and temporal alignment.	Because measurand and timing changed together, their individual contributions cannot be isolated; the 60 min statistical adjustment is not evidence of a physiological transport lag.
Response to experimentally increased circulating-insulin exposure	During euglycemic hyperinsulinemia, serum insulin increased from 176 ± 51 to 36,796 ± 2031 pmol/L, while salivary IRI increased from 253 ± 100 to 1919 ± 437 pmol/L [14]. In insulin-treated diabetes, fasting saliva versus plasma free insulin was r = 0.62 (*p* < 0.001), and the salivary response after subcutaneous regular insulin was displaced by approximately 60 min in the nested dynamic experiment [15].	Salivary insulin can respond to experimentally increased circulating insulin exposure.	Response amplitude and timing are protocol-specific and do not establish proportional or contemporaneous equivalence between matrices.
Temporal correspondence—within-protocol metabolic-state dependence	Within the same OGTT study, approximate peak displacement was ~60 min in healthy participants, ~0 min in T2D, and ~30 min in obese non-diabetic participants [30].	Observed temporal correspondence can vary by metabolic state within a shared broad challenge framework.	No single fixed temporal displacement is supported across these groups; observed displacement should not be interpreted as a physiological transport delay.
Temporal correspondence—across dynamic protocols	Observed salivary displacement was 45–90 min [14], 30–60 min [38], ~30 min [41], and ~30–45 min [12] in different dynamic protocols.	Temporal displacement of the salivary response recurs across several dynamic settings.	These values arise from different populations, protocols, and salivary measurands and are not pooled into a universal lag.
Cross-matrix response amplitude	Within the clamp, serum insulin changed from 176 ± 51 to 36,796 ± 2031 pmol/L, while salivary IRI changed from 253 ± 100 to 1919 ± 437 pmol/L [14]. In a swallowed-meal study, fasting saliva/plasma insulin concentrations were 28 ± 6/40 ± 25 pmol/L, and meal peaks were 96 ± 18/270 ± 66 pmol/L [42].	Both matrices can respond within the same experiment without reproducing the same observed response amplitude.	These within-study observations do not establish a fixed proportional conversion; no derived common ratio is calculated.
Study-specific saliva-to-blood scale	Reported saliva:blood descriptors include 1:1.6 for free insulin [31], salivary insulin approximately 10% of serum insulin [11], fasting saliva:plasma ratios of 1:3.6 versus 1:2.8 by adiposity group [45], and salivary insulin approximately one-half of plasma insulin in the fasting state [12].	A numerical saliva-to-blood scale can be described within specific study conditions.	These descriptors are study specific, are not pooled, and should not be treated as transferable conversion factors.
Explicit quantitative mapping	ln *I_p_* = 0.85 ln *I_s_* + 1.84; R^2^ = 0.67 [44].	An explicit saliva-to-plasma numerical mapping has been reported within a paired source dataset.	Source-dataset fit does not establish calibration, characterize individual prediction error, demonstrate numerical agreement or internal validation, or constitute independent external validation.

Note: AUC, area under the curve; HC, high-carbohydrate condition; IRI, immunoreactive insulin; LC, low-carbohydrate condition; OGTT, oral glucose tolerance test; T2D, type 2 diabetes. I_p_ and I_s_ denote plasma and salivary insulin concentrations, respectively, expressed in pg/mL in the fitted relationship. Quantitative results are retained according to the estimand they directly support and are not pooled across non-equivalent estimands or salivary measurands. Statistical time shifts are not interpreted as physiological transport delays. A study may contribute to more than one evidence cluster when it directly informs distinct estimands.

**Table 3:** Current evidence status for quantitative saliva-to-circulating-insulin inference.

Quantitative Domain	Current Evidence	Evidence Status	Inferential Boundary
Cross-matrix association/tracking	Demonstrated across fasting and dynamic settings, with contextual heterogeneity.	Supported, context-dependent	Supports covariation under defined conditions; does not establish numerical agreement or quantitative substitution.
Temporal correspondence	Temporal displacement has been observed in dynamic studies, with magnitude varying across populations and protocols.	Supported, variable	No fixed universal temporal relationship is established; statistical time shifts do not establish a physiological transport delay.
Scale/proportionality	Study-specific saliva-to-blood scale descriptors and differing cross-matrix response amplitudes have been reported.	Study-specific only	No general proportional scale or transferable conversion factor is established.
Explicit quantitative mapping	An explicit saliva-to-plasma equation has been reported from a paired source dataset.	Demonstrated in one source dataset	Provides a dataset-specific mapping, not a validated general conversion.
Calibration	Calibration of the fixed mapping has not been reported.	Not demonstrated	Source-dataset model fit does not establish calibration.
Individual prediction error	Error for individual circulating-insulin estimates generated from a fixed salivary mapping has not been characterized.	Not characterized	Association or source-dataset model fit does not characterize individual prediction error.
Internal validation of a mapping	Internal validation of the fixed mapping has not been reported.	Not demonstrated	Apparent development-dataset performance does not constitute internal validation.
Numerical agreement/ interchangeability	Direct individual-level agreement has not been assessed.	Not demonstrated	Absence of agreement assessment is not evidence of biological disagreement and does not establish numerical interchangeability.
Independent external validation	The fixed mapping has not been applied unchanged and evaluated in an independent paired cohort without coefficient refitting.	Not demonstrated	Source-dataset performance does not constitute external validation.
Cross-context transferability	Timing, scale, association strength, population, and salivary measurand vary across the available paired studies.	Not established	Quantitative performance cannot be assumed to transfer beyond the contexts in which it has been evaluated.

Note: The quantitative domains are distinct and non-hierarchical, and evidence supporting one domain does not automatically establish another.

**Table 4:** Claim-specific requirements for quantitative validation.

Intended Quantitative Claim	Minimum Requirements for Evaluation	Inference if Requirements Are Met
Cross-matrix association/ tracking	Paired saliva and explicitly specified serum or plasma measurements, with the salivary measurand defined. Association analysis must reflect the participant and repeated-measure structure when applicable [37]. Broader claims require replication beyond the source setting.	Covariation or tracking under the evaluated conditions; not numerical agreement or substitution.
Temporal tracking/ displacement	Temporally resolved paired sampling with sufficient resolution for the intended temporal estimand. The temporal comparison should be pre-specified; any rule derived by exploratory time-shifting should be fixed before subsequent validation.	Temporal correspondence under the evaluated sampling design; not a universal or physiological transport delay.
Contemporaneous estimation	Pre-specified circulating target, salivary measurand, sampling window, units, and prediction rule. Calibration and individual prediction error should be evaluated for the defined contemporaneous target, with validation performed at the participant level [51].	Numerical estimation of the contemporaneous circulating target within the validated conditions.
Stable proportionality/ scale	Commensurate saliva and circulating measurands and units, evaluated across the intended concentration range and conditions. Proportionality must be assessed directly rather than inferred from correlation or isolated ratios.	A defined saliva-to-blood scale only over the range and conditions in which proportionality has been demonstrated.
Quantitative mapping/ calibration	Clearly defined saliva-to-serum or saliva-to-plasma mapping, with predictor definition, reference, transformations, units, measurand, and timing fixed before validation. Calibration and individual prediction error should be evaluated [51].	A calibrated quantitative mapping within the conditions in which its performance has been validated.
Individual prediction	Fixed mapping evaluated at the participant level across the intended measurement range. Individual prediction error and associated uncertainty must be characterized using pre-specified performance measures.	Individual circulating-insulin estimates with characterized error within the validated setting.
Numerical agreement/ substitution	Directly measured circulating insulin and corresponding saliva-derived circulating-insulin estimates expressed on the same quantitative scale. Difference-based agreement should be assessed against a pre-specified acceptance criterion appropriate to intended use, with methods accounting for repeated measurements when applicable [52,53].	Interchangeability only if the pre-specified agreement criterion is satisfied, and only under the validated conditions.
Independent external validation	Independent paired participants who contributed neither to model development nor model selection. The previously specified model should be applied without coefficient refitting, with calibration and individual prediction error assessed and agreement additionally evaluated when substitution is intended [54].	Performance of the fixed mapping in the independently evaluated setting.
Cross-context transferability	Replicated external validation across the populations, physiological conditions, sampling protocols, salivary measurands, and analytical settings encompassed by the intended claim.	Transferability only across contexts in which adequate performance has been demonstrated.

Note: Requirements are claim-specific and non-hierarchical. General principles for model development, internal validation, and external validation follow established prediction-model methodology [54]. When repeated measurements are present, development and evaluation should preserve participant-level dependence. External validation refers to evaluation of a previously specified mapping in independent paired participants before model redevelopment or coefficient refitting. Numerical substitution additionally requires agreement against a pre-specified acceptance criterion appropriate to the intended use.

## Data Availability

No new data were created or analyzed in this study. Data sharing is not applicable to this article.
